# Minimally Invasive Enucleation of Esophageal Leiomyoma: Experience From a Single Institution

**DOI:** 10.7759/cureus.71384

**Published:** 2024-10-13

**Authors:** Tran Phung Dung Tien, Lam Viet Trung, Huan Nguyen Ngoc

**Affiliations:** 1 Digestive Surgery, Cho Ray Hospital, Ho Chi Minh City, VNM

**Keywords:** esophageal leiomyoma, esophageal submucosal tumor, laparoscopic surgery, minimal invasive approach, thoracoscopic surgery

## Abstract

Introduction: Esophageal leiomyoma is a rare disease, most of which are benign, and endoscopic tumor removal surgery is a widely accepted treatment method. The two surgical approaches, laparoscopic and thoracoscopic, must be selected and applied according to the tumor size and location. The study aimed to evaluate the results, feasibility, and appropriate approach of minimally invasive surgery to treat esophageal leiomyoma.

Subjects and research methods: A retrospective study of 27 cases of patients who underwent endoscopic tumor removal surgery to treat esophageal leiomyoma at Cho Ray Hospital was conducted from January 2015 to April 2020. Demographic characteristics, clinical symptoms, surgical characteristics, and postoperative complications were recorded.

Results: A total of 16 men and 11 women were included in the study, with an average age of 47.2 years (25-76 years). In total, 92.6% of patients had symptoms, the most common being dysphagia (n = 19); however, 7.4% of patients still had no symptoms. Tumor removal through right thoracoscopic surgery was performed in 18 patients; the remaining nine patients were approached through laparoscopic intra-abdominal surgery. The median tumor size in all patients is 3.0 cm (2.0-5.0 cm), with the smallest tumor being 1.2 cm and the largest tumor being 8 cm. Postoperative histopathology shows esophageal leiomyoma in 100% of cases. Three cases (11.1%) of esophageal mucosal perforation and one case (3.7%) of tracheal perforation occurred during surgery. There was no case of death or suture leak after surgery. There was one case of postoperative gastroesophageal reflux that was treated successfully with medical treatment for two weeks. The average follow-up period was 21.4 months, with no deaths or recurrences during follow-up.

Conclusion: Endoscopic surgery is a safe, minimally invasive method for esophageal leiomyoma removal, with laparoscopy used for tumors near the gastroesophageal junction and right thoracoscopic surgery for remaining tumors, ensuring quick patient recovery.

## Introduction

Esophageal leiomyomas are mesenchymal tumors of the esophageal wall, present as a heterogeneous group accounting for less than 1% of all esophageal tumors, and the majority are benign [1,2]. Leiomyoma is the most common benign tumor of the esophageal submucosal tumor, accounting for approximately 70%-80% of cases [2]. Other types of tumors, such as gastrointestinal stromal tumors and Schwann cell tumors, are very rare. Diagnosis is difficult because esophageal leiomyoma has clinical symptoms, gastrointestinal endoscopy, and imaging characteristics similar to other benign tumors, so an accurate diagnosis must be based on histology and postoperative immunohistochemistry [3]. Patients with esophageal leiomyomas are mainly between 20 and 50 years old, with more men than women, and this is in the order of 2:1. The occurrence of these tumors in the upper one-third of the esophagus accounts for only 10% of all leiomyomas of the esophagus, and the rest of the tumor location is usually in the lower two-thirds of the esophagus [4].

Tumor excision surgery is widely accepted as a standard treatment method with many surgical approaches, including open and endoscopic surgery and even robotic surgery [5,6]. Surgical treatment is a recommended option because if not treated surgically, the tumor will gradually grow and cause increasing trouble swallowing due to esophageal lumen obstruction, chest pain, potential hemorrhage from mucosal breach, and, in sporadic cases, significant tumor enlargement that may affect adjacent organs. However, some cases of malignant esophageal gastrointestinal stromal tumors may require esophagectomy with higher rates of complications and mortality [3,6-8]. Previously, tumor removal surgery through thoracotomy or laparotomy was often commonly applied [5,6]. Recent reports show the feasibility of removing tumors using endoscopic surgery; however, this technique can be difficult and increase surgical complications in large tumors. We performed this study to evaluate the feasibility, surgical results, and appropriate approach of endoscopic surgery to treat esophageal leiomyoma.

## Materials and methods

A retrospective study of 27 patients who had endoscopic surgery for treatment and histopathological diagnosis after surgery for esophageal leiomyoma was conducted from January 2015 to April 2020 at the Digestive Surgery Department, Cho Ray Hospital. Demographic characteristics, clinical symptoms, surgical characteristics, and postoperative follow-up period were recorded.

Preoperative assessment

All patients received standard evaluations, including chest computed tomography (CT) scans, esophagography, esophagogastroscopy, elective endoscopic ultrasonography, routine blood tests, electrocardiograms, and comprehensive medical questions prior to hospital admission. All of the aforementioned factors could guarantee appropriate surgical indications and exclude patients unable to tolerate the operation.

CT scans pinpoint the esophageal tumor's size, location, and kind. These soft tissue masses are lobulated, smooth, or round and lack mediastinal lymphadenopathy. A single cross-section of a leiomyoma around the esophagus can be observed on a CT scan.

Follow-up

Clinical follow-up information was available for 27 patients for a period ranging from two to 43 months following the initial diagnosis.

Surgical method

The surgical method depends on the location and size of the tumor. We approached the tumor near the gastroesophageal junction using laparoscopic intra-abdominal surgery, while tumors in the remaining locations were approached through thoracoscopic surgery via the right chest. When approaching the tumor through the right chest, everything is done with the patient lying on the left side. To perform endoscopic surgery through the right thoracic cavity, the patient is anesthetized with endotracheal intubation using a double-lumen tube, which allows ventilation of one left lung while the right lung collapses to facilitate surgery. We use three to four trocars for surgery. After determining the tumor location, the mediastinal pleura is cut open along the esophagus, and the muscle layer covering the tumor is opened. The tumor was carefully dissected to avoid perforating the mucosa layer. Insufflation of air into the esophagus through a nasogastric tube is performed to check the integrity of the mucosa after the tumor removal surgery. All patients had the muscle layer sutured after tumor removal to avoid complications of esophageal diverticulum formation after surgery.

Data analysis

Data processing and analysis were performed using IBM SPSS Statistics for Windows, Version 22 (Released 2013; IBM Corp., Armonk, New York) software. The correlation between qualitative variables was evaluated using the chi-square test (with correction according to Fisher's exact test in case the 2 x 2 table has at least one cell with an expectation value of <5). The difference between two quantitative variables was assessed using the t-test for normal distribution and the Mann-Whitney test for non-normal distribution.

## Results

Table 1 presents the patient characteristics. The study included 16 men and 11 women with a mean age of 47.2 years (25-76). There were 13 cases of tumors in the lower third of the esophagus (48.2%), nine cases in the middle third (33.3%), and five cases in the upper. In total, 92.6% showed clinical symptoms (n = 25); only two cases (7.4%) had no symptoms. The tumor was detected through gastroduodenal endoscopy during a routine health check with all patients. The most common symptom was dysphagia (n = 19), major with solid foods. The median tumor size in all patients is 3.0 cm (2.0-5.0 cm), with the smallest tumor being 1.2 cm and the largest tumor being 8 cm. When comparing the median size to find a relationship with clinical symptoms, the median tumor size in symptomatic patients was not significantly different from that in asymptomatic patients (3.0 cm vs. 5.5 cm, p = 0.302).

**Table 1:** Demographic characteristics and clinical symptoms

Characteristics	Values, n = 27 (%)
Age	47.2 (25-76 years)
Gender	-
Male	16 (59.3%)
Female	11 (40.7%)
Esophageal tumor site	-
Upper	5 (18.5%)
Middle	9 (33.3%)
Lower	13 (48.2)
Symptom	-
No symptom	2 (7.4%)
Dysphagia	19 (70.4%)
Epigastric pain	7 (25.9%)
Chest pain	2 (7.4%)
Pain behind the sternum	1 (3.7%)
Heartburn, belching	1 (3.7%)
Tumor size (cm)	-
All patients	3.0 (2.0-5.0)
No symptom	5.5 (3.0-8.0)
Present symptom	3.0 (2.0-5.0)

Thoracoscopy was performed through the right chest on 18 patients, while the remaining nine patients underwent laparoscopic intra-abdominal tumor removal surgery. There were three cases (11.1%) of mucosal perforation during surgery. In case a perforation of the mucosa is detected during surgery, the mucosa will be sutured and then checked by injecting air into the nasogastric tube to confirm that the mucosa has been sutured closed. All patients were given sugar water on postoperative day one and liquid food on postoperative days two and three. The patients were then given solid food on day four. There were no cases of leakage after tumor removal. One case (3.7%) of tracheal perforation occurred during the removal of a 7 cm leiomyoma located in the middle one-third of the esophagus, which was firmly attached to the base of the left bronchus (more details in Table 2). During the process of removing the tumor, the main bronchus was punctured. The left main bronchus perforation was then sutured, the patient was healthy postoperatively, a straight chest X-ray showed no pneumothorax, the pleural drainage tube was removed on the seventh postoperative day, and the patient was discharged on postoperative day eight. There were no significant postoperative complications. The average postoperative hospital stay is 5.2 days (three to eight days).

**Table 2:** Surgical characteristics and postoperative complications

Characteristics and Complications	Values, n = 27 (%)
Thoracoscopy	18 (66.7%)
Laparoscopic intra-abdominal	9 (33.3%)
Operating time (minute)	146.5 ± 37.5 (90-210)
Mucosa perforation	3 (11.1%)
Tracheal perforation	1 (3.7%)
Leakage	0 (0%)
Postoperative time (day)	5.22 (3-8)

The average follow-up time was 21.4 months (two to 43 months). During follow-up, all patients were required to undergo esophagogastroscopy once after one month and then once every 12 months. We did not perform chest CT scans or endoscopic ultrasound (EUS) because no lesions were seen during esophagogastroscopy for all patients during the follow-up period. There is one patient who developed symptoms of gastroesophageal reflux two weeks after surgery to remove a leiomyoma in the lower third of the esophagus despite having no prior symptoms and showing improvement following medical treatment. All cases of esophageal leiomyoma had no deaths, no recurrence, and no long-term complications.

## Discussion

Esophageal leiomyoma was initially documented by Virchow in 1867, and the first successful surgical excision of this tumor was reported by Sauerbruch in 1932 [9]. Leiomyomas are rare mesenchymal tumors, present in less than 1% of all esophageal tumors, and the histopathology is highly variable. Although tumors can cause symptoms such as choking, epigastric pain, and reflux, they can be accidentally discovered without symptoms. According to the results of surgery to remove esophageal submucosal tumors in 87 cases reported by Shin et al., 66.7% of cases were asymptomatic, and symptomatic patients had significantly larger tumors compared to asymptomatic patients [10]. The diagnosis of esophageal submucosal tumors must be based on postoperative histopathology. Bonavina et al. recommended against performing endoscopic biopsy of esophageal submucosal tumors because endoscopic biopsy can inflame or damage the mucosa, which may increase the risk of mucosal perforation during tumor removal [5]. We do not routinely perform endoscopic biopsy if the submucosal tumor has a benign phenotype on a CT scan or EUS. However, in many cases, it is difficult to distinguish between benign and malignant tumors on EUS, so a biopsy will be performed in cases where the tumor has a phenotype that does not exclude malignancy with a higher probability. In our study, endoscopic tumor removal surgery was approached through the right chest and abdomen, even with tumors as large as 8 cm without any significant complications, with a median postoperative hospital stay of only 5.2 days.

There are many treatment options for esophageal leiomyomas, such as surveillance, therapeutic gastrointestinal endoscopy, and surgical treatment [6]. Surgery is a treatment option for esophageal leiomyomas, and tumor excision is widely accepted as a standard treatment [5,6]. Open surgery to remove the tumor was a popular method in the past [5,6]. Since Everitt et al. performed endoscopic removal of esophageal submucosal tumors in 1992, thoracoscopic surgery has been increasingly used, and several studies have examined its feasibility and safety [11-16]. Research by von Rahden et al. found that, compared with open surgery, endoscopic removal of esophageal submucosal tumors reduces lung complications, hospital stay, and postoperative pain [15]. Kent et al.'s study also showed that endoscopic surgery had a shorter hospital stay when compared with the open surgery group [13]. In our study, the average hospital stay after surgery was only 5.2 days.

There is no consensus regarding the indications for endoscopic tumor removal. Jiang et al. suggested that esophageal leiomyomas measuring 1-5 cm are ideal for endoscopic transthoracic tumor removal surgery [12]. Tumors larger than 5 cm can also be removed endoscopically, although this may increase the likelihood of conversion to open surgery [13]. In our study, we performed thoracoscopy surgery to remove the tumor through the right chest, with the largest tumor size being 8 cm, without having to convert to open surgery or have any serious complications. The conversion rate to open surgery is 0%. All cases of mucosal perforation during tumor removal were sutured, and all patients were discharged without complications.

The follow-up period after surgery for benign esophageal submucosal tumors is not specific. Recurrence of remaining esophageal submucosal tumors is rarely reported, so they may not require oncological follow-up. Instead, postoperative digestive function, such as postoperative gastroesophageal reflux, should be considered. Kent et al. reported the results after tumor removal in 20 cases of esophageal submucosal tumors; five cases required surgery to create an anti-reflux valve after surgery due to reflux symptoms or worsening reflux symptoms [13]. In our study, only one patient developed new reflux symptoms after surgery and received good medical treatment with proton pump inhibitors for two weeks; no deaths or recurrence occurred during the follow-up period.

Limitations of the study

This is a retrospective study with a small sample size, conducted at a single center, and with an insufficient follow-up period, resulting in many limitations. Multicenter studies, larger sample sizes, and longer follow-up periods are needed to better determine the recurrence rate and complications of surgery.

## Conclusions

Tumor excision surgery is a necessary method to treat esophageal leiomyoma. An endoscopic surgical approach is feasible, effective, and safe for selected patients, resulting in short hospital stays and rapid patient recovery. Laparoscopic intra-abdominal surgery should be applied to tumors near the esophageal-gastric junction. Thoracoscopy surgery through the right chest should be performed to remove the tumor in accordance with the remaining position of the esophagus. Oncological follow-up is unnecessary for leiomyomas due to the minimal recurrence rate, but postoperative gastrointestinal function should be monitored.
